# Supplementary material for: Functional connectivity between the amygdala and prefrontal cortex underlies processing of emotion ambiguity
Source: Transl Psychiatry. 2023 Oct 28;13:334. doi: 10.1038/s41398-023-02625-w (PMC10613296; doi:10.1038/s41398-023-02625-w)
Supplement: Supplementary file 1 — Supplemental Material [file 41398_2023_2625_MOESM1_ESM.pdf]

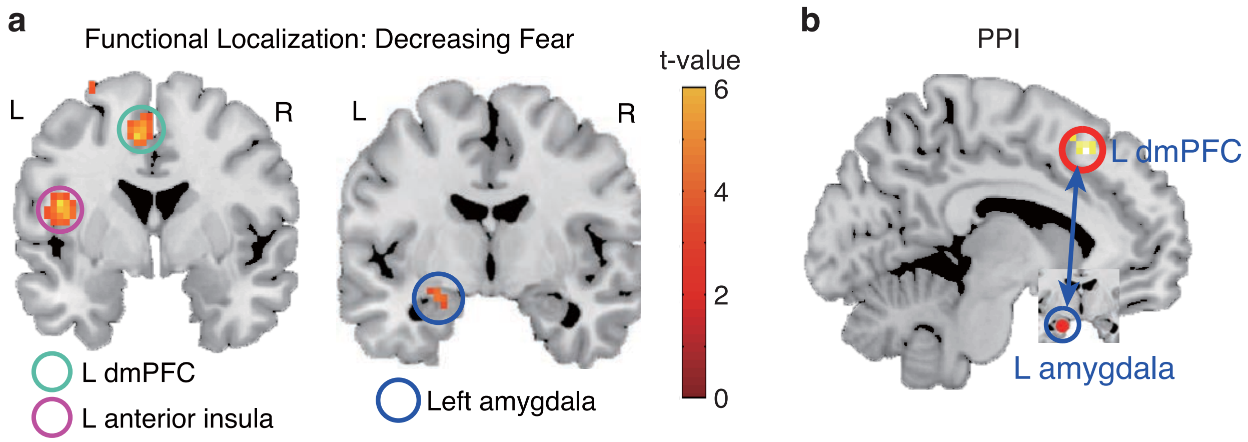

Supplementary Fig. 1. Psychophysiological interaction (PPI) analysis for emotion (i.e., fear) intensity. **(a)** Decreasing fear intensity was correlated with increasing BOLD activity in the left dorsomedial prefrontal cortex (dmPFC), left anterior insular, and left amygdala. **(b)** PPI analysis revealed functional connectivity between the amygdala and dmPFC (peak: $x = -9$, $y = 24$, $z = 48$; 13 voxels, SVC, FWE P = 0.05).

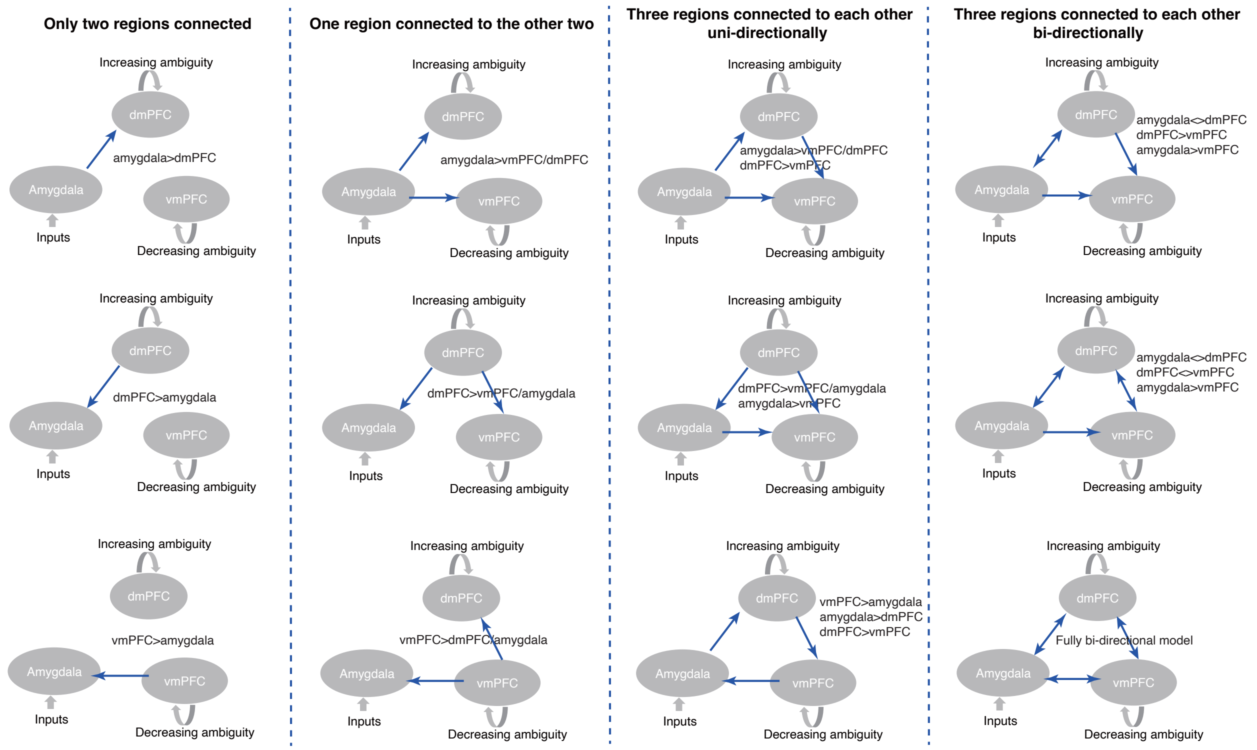

Supplementary Fig. 2. A list of dynamic causal modeling (DCM) models that were analyzed in the present study.

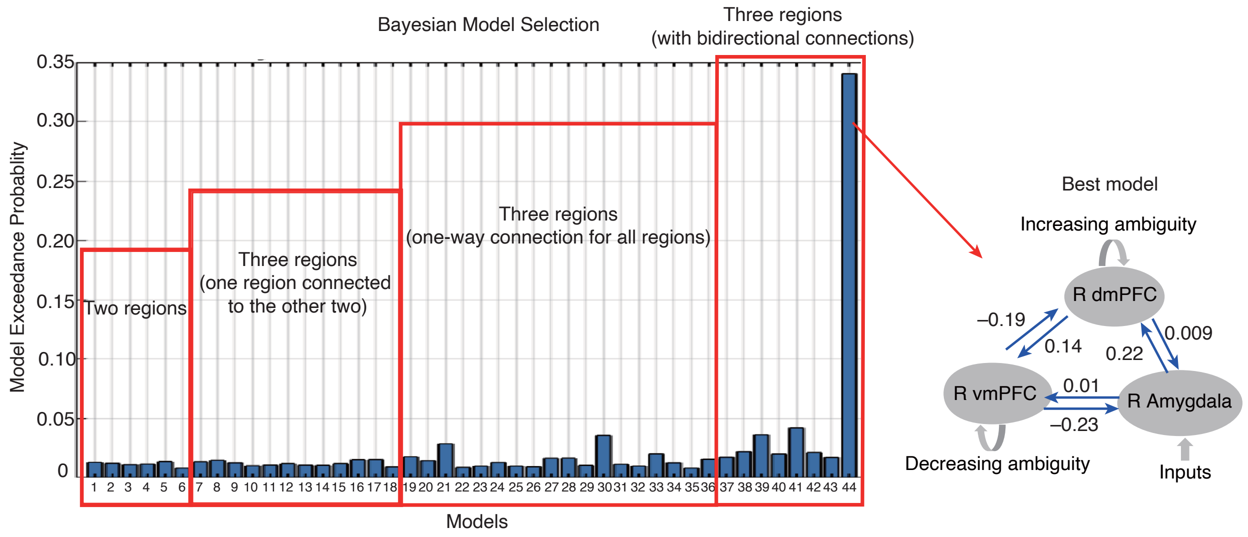

Supplementary Fig. 3. Control DCM analysis with the right dorsomedial prefrontal cortex (dmPFC) and ventromedial prefrontal cortex (vmPFC). Legend conventions as in **Fig. 2**.

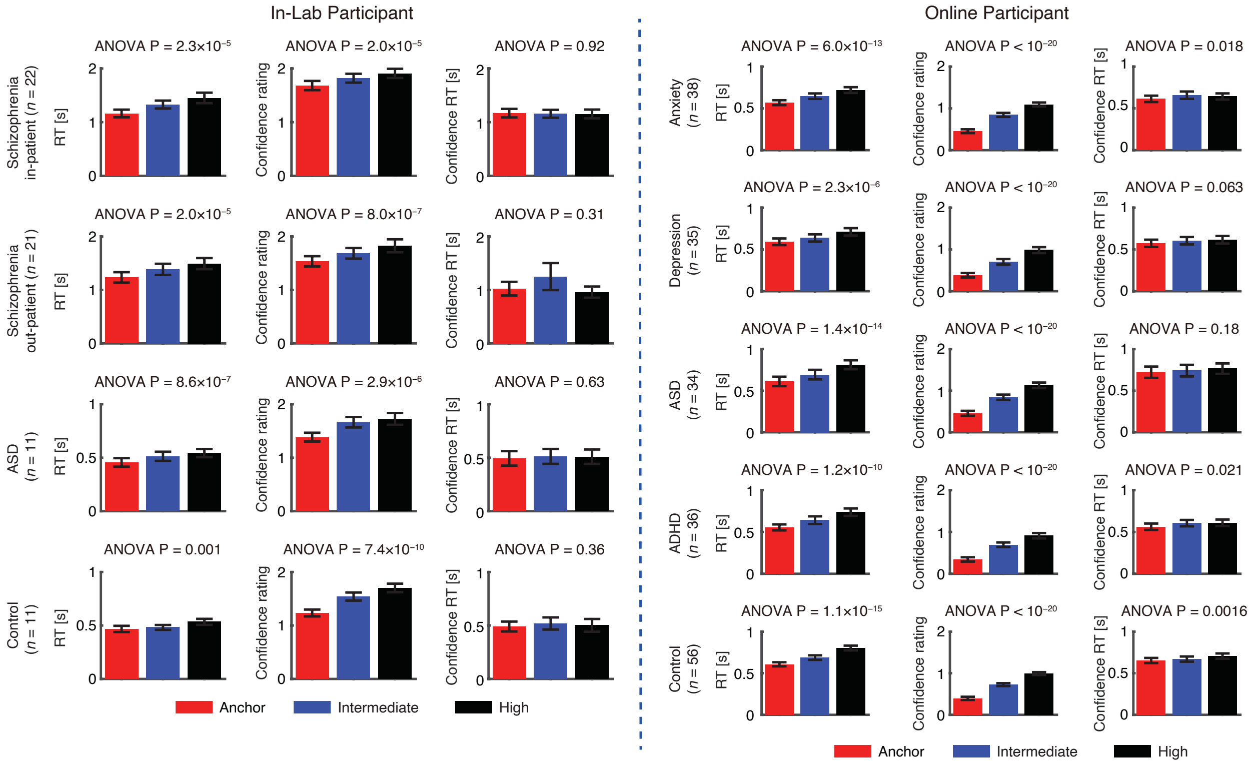

Supplementary Fig. 4. Reaction time (RT) and confidence rating (CR) for each condition and each participant group. Error bars denote ±SEM across participants. Shown above each plot are the p-values from one-way repeated measures ANOVA across conditions.
